# Space Use and Movement of Urban Bobcats

**DOI:** 10.3390/ani9050275

**Published:** 2019-05-24

**Authors:** Julie K. Young, Julie Golla, John P. Draper, Derek Broman, Terry Blankenship, Richard Heilbrun

**Affiliations:** 1 USDA National Wildlife Research Center, Millville Predator Research Facility, Logan, UT 84321, USA; 2 Department of Wildland Resources, Utah State University, Logan, UT 84322, USA; jmgolla@gmail.com (J.G.); john.draper@usu.edu (J.P.D.); 3 Oregon Department of Fish & Wildlife, 4034 Fairview Industrial Drive SE, Salem, OR 97302, USA; derek.j.broman@state.or.us; 4 Welder Wildlife Foundation, Sinton, TX 78387, USA; tblankenship@welderwildlife.org; 5 Government Canyon State Natural Area, Texas Parks and Wildlife Department, San Antonio, TX 78254, USA; richard.heilbrun@tpwd.texas.gov

**Keywords:** *Lynx rufus*, anthropogenic resources, carnivore, behavior, spatial ecology

## Abstract

**Simple Summary:**

The bobcat (*Lynx rufus*) is a medium-sized carnivore that lives in remote and urban habitats. Here, we evaluate how bobcats exploit a highly urbanized section of the Dallas–Fort Worth metroplex, Texas, USA by evaluating their space use and activity patterns. We found that bobcats use more natural habitat areas within urban areas, such as agricultural fields and creeks, and avoid highly anthropogenic features, such as roads. Bobcat home ranges overlap one another, especially in areas with preferred habitat types, but they are neither avoiding nor attracted to one another during their daily movements. This study highlights how bobcats are able to navigate a built environment and the importance of green space in such places.

**Abstract:**

Global urbanization is rapidly changing the landscape for wildlife species that must learn to persist in declining wild spacing, adapt, or risk extinction. Many mesopredators have successfully exploited urban niches, and research on these species in an urban setting offers insights into the traits that facilitate their success. In this study, we examined space use and activity patterns from GPS-collared bobcats (*Lynx rufus*) in the Dallas–Fort Worth metroplex, Texas, USA. We found that bobcats select for natural/agricultural features, creeks, and water ways and there is greater home-range overlap in these habitats. They avoid roads and are less likely to have home-range overlap in habitats with more roads. Home-range size is relatively small and overlap relatively high, with older animals showing both greater home-range size and overlap. Simultaneous locations suggest bobcats are neither avoiding nor attracted to one another, despite the high overlap across home ranges. Finally, bobcats are active at all times of day and night. These results suggest that access to natural features and behavioral plasticity may enable bobcats to live in highly developed landscapes.

## 1. Introduction

Human populations are expanding alongside anthropogenic development and loss of natural landscapes. At a global scale, carnivore populations and their large prey species have declined [1]. Contributions to their decline include both indirect impacts, such as habitat loss and fragmentation [2,3], and direct impacts, such as retaliatory killing or hunting [4]. Carnivores are often a source of conflict with humans because they may kill livestock and therefore impact livelihoods [5] or are viewed as a real or perceived threat to human health and safety [6,7]. Thus, carnivores must learn to live in the remaining and ever-dwindling natural landscapes or adapt to survive in anthropogenic landscapes.

Some of the most densely human-populated places in the world are also home to thriving populations of carnivores. Research has begun to focus on carnivores living in anthropogenic landscapes, with the most recent work focused on highly urbanized landscapes. Established populations of leopards (*Panthera pardus*) and striped hyenas (*Hyaena hyaena*) live amidst densely populated rural and urban areas of India [8,9,10]. Spotted hyenas (*Crocuta crocuta*) live entirely on domestic prey and anthropogenic scraps in Ethiopia [11,12] and benefit from increased human activity and presence in Kenya [13]. Mountain lions (*Puma concolor*) in the western United States persist in patches of highly fragmented urban landscapes [14]. Coyotes (*Canis latrans*) are well documented in urban habitats across North America and are considered one of the most urban-adaptable carnivore species [15]. Even bobcats (*Lynx rufus*), previously considered a disturbance-sensitive species [16], have been shown to persist in suburban, fragmented, and developed areas [17,18,19,20,21].

Many of the carnivore species now living in urban areas exhibit territoriality in their native landscapes. In urban areas, they live at higher densities and on smaller home ranges [22]. A territory must include enough space for individuals or packs within each occupied range to meet foraging, refuge, and reproductive needs while excluding conspecifics from access to the resources in that defined space [23]. Thus, the very nature of territoriality could affect a species’ ability to reside in an urban landscape. Carnivores in urban areas have adapted to life in anthropogenically transformed landscapes. Such adaptations to existing at high densities with greater overlapping space use may also influence other behavior, such as territorial behavior and daily activity patterns [17,19,24,25]. Alternatively, in some cases, territorial behavior may be maintained or amplified, as smaller spaces could be easier to defend and increased competition for space or other resources would justify more territorial defense. To date, most studies of urban carnivores have focused on peri-urban or suburban areas and little information is available from epicenters within major metropolitan areas that could add clarity to territorial behavior (but see [15,26] and references therein).

Our study sought to learn more about bobcat spatial ecology in the context of an established urban population. We focused on bobcats because they are historically known to be elusive, sensitive to human disturbances, and uncommon in areas densely populated by humans [16]. However, some bobcat populations exist in a peri-urban setting within close proximity to or overlap with areas of high human densities [17]. Bobcats are mostly solitary except during mating and when females are raising young [26,27]. They typically occupy consistent home ranges over time, established via a land-tenure system and maintained by scent marking [26,28,29,30,31,32]. Home ranges are well defined with limited overlap [27], especially of core-use areas [33]. Aggressive defense of home ranges has been documented [32]. They are considered obligate carnivores and rely on a diet of live prey such as small mammals and birds [34], which may be abundantly available and with fewer antipredator defense strategies in urban settings [35]. Past bobcat studies have followed the trajectory of most other studies on carnivores, which often focus on natural landscapes and more recently highlighted populations along the urban–wildland interface (e.g., [36]). Only recently has attention focused on understanding bobcat ecology in more urban areas [18].

We aimed to understand to what extent bobcats utilize highly developed habitat in a major metropolitan area. The specific objectives were to determine habitat selection, space use, home-range overlap, and activity patterns of bobcats in the Dallas–Fort Worth (DFW) metroplex, Texas, USA. Results of this study can help inform management and conservation of carnivores within urban landscapes.

## 2. Materials and Methods

### 2.1. Study Site

The DFW metroplex, Texas, USA is the 4th largest, 3rd fastest growing, and 19th most densely populated metropolis in the United States (U.S. Census Bureau 2014). It covers more than 24,000 km^2^ of rolling hills and large floodplains that require extensive networks of storm drainages and creeks to divert flood water into the Trinity River. The metroplex lies within the Cross Timbers area of Texas, formerly covered in habitat types such as oak trees (*Quercus* sp.) and Backland Prairie [37], and is now a mosaic of urban structures which includes retail stores, residential areas, city parks, golf courses, and patches of undevelopable wetland, connected by a network of roads, highways, and interstates.

**Figure 1:**
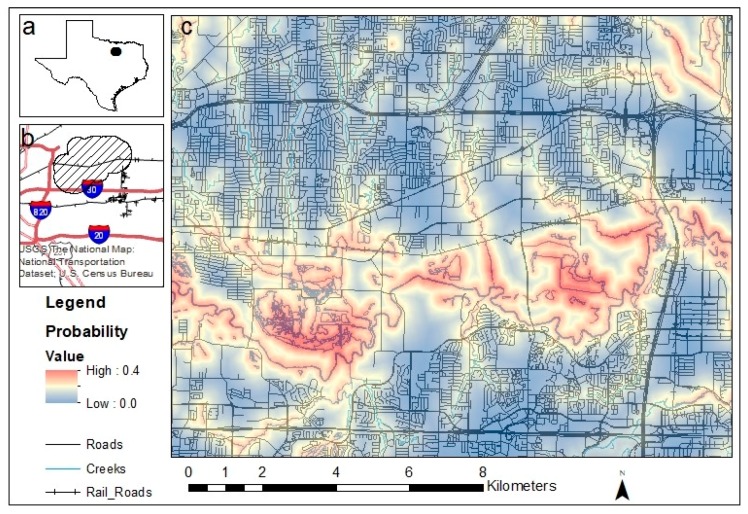
(**a**) Location of the Dallas–Fort Worth (DFW) metroplex, Texas, USA; (**b**) specific study site (hashed polygon) within the DFW metroplex; and (**c**) heat map from the resource selection function model created using data from GPS-collared bobcats in the study site. The Trinity River runs east–west through the south-end of the study area.

The study area was in the center of the DFW metroplex, bordered by state highways, an interstate, and the west fork of the Trinity River (Figure 1). The area covers approximately 78.1 km^2^ and includes developed parts of Fort Worth, Arlington, Hurst, Bedford, Euless, and Grand Prairie municipalities. The site was selected based on its high frequency of bobcat sightings by the public prior to our study and complete immersion within dense urban development. The area includes several types of urbanization such as interstate rights of way, railroad tracks, industrial zones, residential neighborhoods, retail areas, developed open space, narrow linear patches of natural habitat (i.e., neighborhood greenbelts), and larger patches of green space within the Trinity River riparian corridor.

### 2.2. Captures

We captured and handled bobcats in accordance with the National Wildlife Research Center’s Institutional Animal Care and Use Committee (IACUC) regulations (QA-2211). We used modified live cage traps (Tomahawk Live Trap Co., Tomahawk, WI, USA; 107 × 38 × 51 cm) to capture bobcats and immobilized captured individuals with a weight-appropriate dose of 5:1 ketamine (10 mg/kg)–xylazine (0.75 mg/kg) mixture sedative [38]. We collected body measurements, blood, hair, parasites, and pelage photos [39] for physiological data on each captured bobcat for this study and as part of broader project goals. A tooth was removed from adult bobcats (*n* = 9) to validate visual inspection used for aging. Initial trapping occurred from January to April 2014, with targeted recapture attempts occurring from September to December 2014 and in March 2015 for bobcats with GPS collars that did not drop off as expected. We typically placed trap sites along natural corridors with probable bobcat traffic but limited human traffic.

### 2.3. GPS Collars and Location Data

We fitted bobcats weighing >5 kg with Lotek (Newmarket, ON, Canada) 3300 s store-on-board GPS tracking collars equipped with drop-off mechanisms. This weight was necessary to ensure the collar was <3% the body weight of the bobcats. We programmed GPS collars to record a GPS location every 2 h from 6:00 p.m. to 6:00 a.m. (nocturnal activity) and every 4 h from 6:00 a.m. to 6:00 p.m. (diurnal activity) with some variability in time. These settings were chosen in order to maximize data collection in consideration of known bobcat activity patterns suggesting they would be more active between dusk and dawn [16,40] and the expectations of collar battery life. We wanted to be able to get at least a full year of data to capture all seasons. In addition to this daily data collection, we programmed collars to record locations at 20 min intervals for a 6 h time period on the 15th of each month, which was used only to obtain finer-scale information on activity patterns. For each collar, this would occur across one of four randomly chosen 6 h time periods. The drop-off mechanisms were programmed at 56 weeks after collar deployment, allowing for just over one year of data collection.

We removed location data within the initial 24 h of data collected after an animal was collared to allow for full recovery time from immobilization drugs administered during captures. We also excluded bobcats with less than six months of GPS-collar data from analysis to ensure sufficient data for home range estimation. Further, points that had a dilution of precision (DOP) > 5.0 and those that failed to obtain a 3D fix were removed to increase spatial accuracy [41]. We then created point files in ArcGIS 10.2.0 (Esri, Redlands, CA, USA) [42].

### 2.4. Home Range and Habitat Analyses

We estimated home ranges for each bobcat utilizing 95% kernel utilization distribution (KUD) for use in both evaluation of home-range overlap and resource selection models [43,44]. Home-range estimates were calculated using the adehabitatHR package in Program R [45,46]. KUD home-range estimates were used for resource selection function analyses described below.

To compare home-range estimates with previous studies and account for the hard boundaries and patchy nature of our urban landscape [47,48], we calculated 99% local convex hulls (LoCoH) using the rhr package in Program R [46,49]. For valid comparison to results from previous studies, we only removed locations with a dilution of precision value of >8 for this particular home-range analysis but otherwise followed the location protocol described above. The Local Convex Polygon Tool was used to produce type “a”, “k”, and “h” LoCoH polygons, considering both 0.99 and 0.95 quantiles in each. In evaluating outputs, the 0.99 type “a” local convex hull was most appropriate in representing bobcat home ranges amidst the patches and barriers of the DFW landscape.

We compared home-range overlap using the 95% KUDs estimated for habitat selection. Percentage of overlap was calculated using adehabitatHR [45] for each individual pair. We then ran linear models to evaluate whether an individual’s age, sex, or the difference in age between two individuals affected how much a bobcat’s home range overlapped the other or was overlapped by the other. We also compared habitat variables and relative selection strength, described below, between the area of home-range overlap and the nonoverlapping areas of all home ranges.

We imported and prepared habitat layers in ArcGIS 10.5.1 [42]. The habitat layers used included the 2011 National Land Cover Database (NLCD) [50] and layers for creeks (2012), railroads (2014), and roads (2011) from the Texas Natural Resources Information System (TNRIS) [51]. Once imported, we clipped all data to the study area to allow for manageable file sizes. All layers were then imported into R, where nonraster layers were converted to 30 m raster cells to match the NLCD data. All layers were then processed to calculate “distance to” features and a raster containing these values was created.

We created a correlation matrix for all features. All variables with a Pearson’s correlation coefficient > 0.7 were grouped together or considered separately to evaluate which was more informative. All the values grouped together were from the NLCD dataset as follows: the three levels of development intensity and developed open space became a single category for developed space, and all of the various natural or agricultural variables were combined into agricultural/natural. Distance to a road from the TNRIS road layer showed a high level of correlation with the developed category. Both were used in the analysis but never in the same model. We standardized all habitat variables to aid in model convergence.

We next extracted habitat variables for each collar location that fell within the 95% isopleth of a given individual’s locations. Binomial generalized linear models were formulated to compare used versus available habitat utilizing individual use and availability as random effects [52]. Global models were formulated using the developed or the distance to roads variables to represent development density. We evaluated stepwise simpler models and determined the top model utilizing AICc values [53]. The top model was then used to generate a raster of relative selection strength across habitats in the study area [54].

### 2.5. Spatial Proximity and Activity Analysis

To assess simultaneous space use, we conducted a proximity analysis (Prox) [55] with multiple distance thresholds using the wildlifeDI package in Program R [46,56]. Prox measures the proportion of time that two individuals are within a certain distance threshold of each other simultaneously (we set the threshold to <5 min because our collars were on the same fix schedule). Only pairs that showed ≥0.01 at the 1500 m threshold (i.e., the minimum distance between home ranges that do not abut or overlap each other) were included to ensure only adjacent pairs were considered. We then modeled the effect of the difference in the pair’s age and sex pairing (male–male, male–female, and female–female). We also ran a nonsimultaneous dataset randomly drawn for each pair’s location data through a Prox analysis for comparison to the simultaneous Prox values. This allowed us to assess if individual bobcats were temporally partitioning their home ranges and only utilizing overlapping portions while the other was farther away. Lastly, we used the 20 min interval data from GPS collars to run categorical linear regressions. For this, data were binned by the hour to identify if there was daily variation in rates of movement.

## 3. Results

### 3.1. Bobcat Captures

We captured 17 bobcats, 12 of which met weight requirements and were therefore fitted with GPS collars (male = 7, female = 5). Of the 12, 9 (male = 5, female = 4) were aged as adults and 3 (male = 2, female = 1) as juveniles. We successfully collected 10 of the GPS collars from the 12 that were deployed. We could not find one collared juvenile male bobcat despite extensive ground and aerial searches, so it was unclear if the GPS collar failed or the bobcat dispersed from the study area, and we were unable to recapture an adult male bobcat to remove a collar that failed to drop off. Of the 10 retrieved collars, three were deployed for the full 56 weeks and seven were deployed for 7–46 weeks (average = 35.0, SE = 6.5). Fix rate success averaged 77% (SE = 2.4). Following the screening, 17,918 GPS locations from nine bobcats (average = 1791.7, SE = 348.9) were used for home-range analysis using local convex polygons and 12,740 GPS locations from nine bobcats were used for home-range analysis using KUD. The bobcat with only seven weeks of data was removed from analysis. When noted, the juvenile male bobcat determined to be dispersing while wearing a GPS collar was also excluded from analyses. Thus, sample size varies between eight and nine and is, therefore, provided throughout the results for clarity.

### 3.2. Home Range Analysis

The 95% kernel-density estimation (KDE) of home-range sizes for GPS-collared bobcats ranged between 2.0 and 17.9 km^2^. The average home-range size was 7.6 km^2^ (SE = 1.7, *n* = 9). When we only considered resident bobcats, home ranges were between 2.0 and 10.9 km^2^, with an average of 6.3 km^2^ (SE = 1.2, *n* = 8). Home ranges of males were on average 10.2 km^2^ (*n* = 5) and the average home-range size for females was 5.5 km^2^ (*n* = 5). The LoCoH estimator resulted in an average home range size of 3.8 km^2^ (SE = 0.81, *n* = 9) for all resident bobcats, 2.3 km^2^ (SE = 1.03, *n* = 5) for resident females, and 5.1 km^2^ (SE = 1.13, *n* = 3) for resident males. The dispersing male bobcat had the largest home range (26.0 km^2^) and was not included in calculating these home range averages.

**Figure 2:**
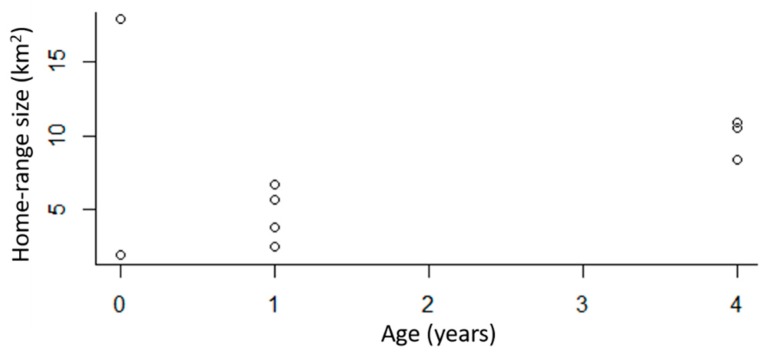
The 95% kernel-density estimation (KDE) home-range estimates for GPS-collared bobcats (*n* = 9) in the DFW metroplex, Texas, USA. This includes the home-range estimate for one dispersing juvenile.

When we removed the dispersing male bobcat, there was a difference in home-range size based on age (β = 1.86, SE = 0.35, *p* = 0.0019, *n* = 8), with older bobcats having larger home ranges than younger bobcats (Figure 2). Home-range overlap also varied by age, with older bobcats overlapping other bobcats more than younger bobcats (β = 0.11, SE = 0.03, *p* = 0.004, *n* = 9). The results were similar if we excluded the juvenile male that was categorized as a disperser (β = 0.12, SE = 0.03, *p* = 0.002, *n* = 8). There was no variation in home-range overlap by sex (*p* = 0.1, SE = 0.33, *n* = 9).

### 3.3. Habitat Analysis

Habitat types within the area of home-range overlap were significantly different from home-range habitat that did not overlap with other bobcats. Overlap habitat was 184 m further from roads (SE = 1.84, *p* < 0.001), 519 m closer to agricultural/natural habitat types (SE = 5.099, *p* < 0.001), and 346 m closer to creeks or water (SE = 2.09, *p* < 0.001) relative to habitat areas without overlap. As would be expected, relative selection strength was higher within the area of overlap; the probability of selection was 0.19 within overlap, making it almost double the 0.1 probability of selection outside of it (SE = 0.00006, *p* < 0.001).

The top resource selection model included the variables representing distance to roads, creeks, and agricultural/natural spaces. Bobcats showed an avoidance of roads (β = −0.14, SE = 0.01, *p* < 0.001) and an affinity for the agriculture/natural habitat type (β = −0.17, SE = 0.23, *p* < 0.001). The strongest effect was an affinity for creeks and water (β = −0.48, SE = 0.01, *p* < 0.001). The predicted relative habitat use for the study area ranged from 0.0 to 0.4 (Figure 1).

### 3.4. Spatial Proximity and Activity Analyses

**Figure 3:**
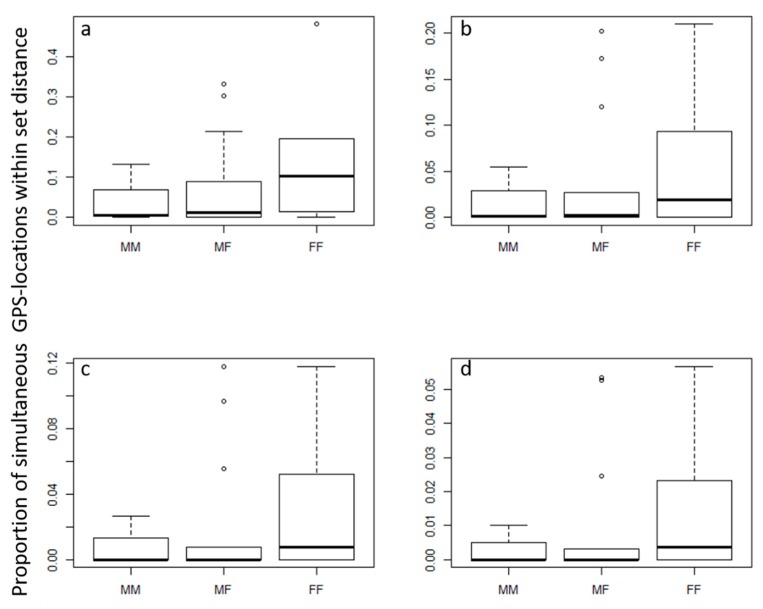
Proportion of simultaneous GPS locations for male–male (MM), male–female (MF), and female–female (FF) bobcats that were simultaneously within (**a**) 1000, (**b**) 700, (**c**) 500, or (**d**) 300 m of one another.

We found no significant variation between simultaneous location data of bobcat pairs due to the difference in age or sex pairing using Prox analysis (Table 1). The same results were observed when the juvenile male that was categorized as a disperser was removed from the analysis (Table 1). Further, the juvenile disperser showed no difference in proximity patterns with resident cats than resident cats had with each other. However, female–female pairings showed much higher variability in tolerance for overlap, while male–male pairs showed very narrow and low tolerance (Figure 3). There was also no difference in the proportion of proximate locations when compared to randomly generated locations that were not simultaneous (Table 1). Finally, there was minor variation in the rates of movement throughout the day (*p* < 0.0001, R^2^ = 0.02), with slightly reduced activity between 6:00 a.m. and 4:00 p.m. and slightly increased activity between 4:00 and 10:00 p.m. There was no observed partitioning of active periods between neighbors.

## 4. Discussion

Urban systems allow for a species that typically has larger area requirements and well-defined home ranges to use different life history strategies [22]. Unless terrestrial carnivores are able to adjust to changes in resource availability, their ability to survive in an urban landscape may be reduced. This is exemplified in our study, where bobcats had small home-range sizes and high overlap with neighbors. They avoided roads and showed the least amount of home-range overlap in road habitat but were attracted to creeks and water and natural/agricultural areas and showed greater overlap in these habitats. Older bobcats in the DFW metroplex had a larger home-range size and greater home-range overlap, suggesting that as bobcats become more established in the space they use, they adapt to spatiosocial characteristics driven by urban landscapes. Alternatively, older cats may have a dominant social status that facilitates access to more space than possible for younger cats. These results suggest that urban bobcats seek natural features and will use developed features, but there is a threshold for the types of developed habitats that are tolerated. Unlike previous studies that suggest that bobcats negatively respond to highly developed urban habitats [57,58], our results show that bobcats will use highly urbanized habitats by taking advantage of natural habitat areas.

Home ranges for both sexes were relatively small [30] but similar to home-range sizes previously reported in other urban areas [17]. The similarities between home-range sizes of DFW bobcats and other peri-urban bobcat populations are interesting to note despite the differences in the degree of urbanization and the resulting size of preferred habitat patches available to bobcats within each study site. Bobcats in our DFW study site lived in the epicenter of a highly urban landscape. Many urban landscapes in southern California occur in mountainous terrain, where areas that are too steep or unstable to develop remain as natural habitat fragments for urban wildlife [17]. Undeveloped areas within DFW are smaller and generally associated with linear floodplains and wetlands interspersed through the urban matrix. However, we note that different studies used different sampling designs and analyses, so we cannot entirely rule out that home-range sizes are not similar. We reported home-range estimates from two estimators to allow for direct comparisons.

Home-range overlap was consistent with some previous bobcat studies suggesting high overlap of bobcats in urban areas [17]. However, our results related to overlap represent a minimum amount of overlap since it is likely that other territorial bobcats occupied our study site, as we repeatedly detected some uncollared bobcats on camera traps [21]. We have no reason to suspect the collared bobcats used space differently from uncollared bobcats and these results still provide insight, albeit with this caveat, into how bobcats utilize shared urban space. Even so, we found differences in overlap among pair types to previous studies. In our study, bobcats that were older were more likely to overlap with other bobcats but there was no relationship between sex and home-range overlap. While we could not find information by age from previous studies beyond age category, several studies have reported differences in intrasexual overlap. Two studies have shown higher percentage overlap among male–male home ranges [59,60], while other studies reported no male–male overlap but considerable overlap among females [61]. Still other studies suggest relatively high levels of intrasexual home-range overlap for males and females [33,62]. The ambiguity in results suggest intrasexual social tolerance, as expressed via home-range overlap, is relatively plastic in bobcats and based on local conditions.

Indeed, home-range overlap relates to a combination of the limited amount of natural space, reduced dispersal opportunities for resident bobcats [63], and the abundance of prey items in urban areas [64,65]. We found bobcats were more likely to overlap in areas with more natural habitat features. As we predicted, resource selection function (RSF) models showed they also generally selected for areas closer to more natural cover, such as natural/agriculture and creeks and waterways, and most avoided areas close to roads. This was similar to, but not as strong as, an association found in a much smaller urban area of Texas. There, Lombardi et al. [18] found peri-urban bobcats primarily associated with areas of green space. Poessel et al. [66] studied heavily urbanized bobcats and, like in our study, noted bobcats avoided roads. We observed bobcats using culverts and other underpasses to cross roads, but this was likely related to their use of another linear feature, creeks. Creeks were an important variable in our study system; they are abundant on the landscape both in natural and developed areas within the DFW study site. When bobcats use creeks in urban areas, they act as narrow corridors amidst urban development [20]. This was apparent upon visual inspection of our heat maps, where the highest probability of selection areas followed the Trinity River corridor (Figure 1). This is also ecologically intuitive; bobcats are known to utilize edge habitats for travel and foraging [16]. Yet, these high probability of use areas include developed sections, as even intermediate distances from most of the creeks on the landscape fall within highly developed areas that were otherwise avoided. Creeks and associated landscapes in urban areas may be altered in the future due to climate change (e.g., [67]), impacting their utility to bobcats and other urban wildlife. These riparian areas will continue to be important to bobcats and other wildlife and are also major factors in urban design due to their role in floods/storm water management and wetland mitigation.

Bobcats may seek these natural areas within a highly developed landscape because they are likely to also have a high density of prey items supported by diverse plant assemblages and anthropogenic food and habitat (e.g., bird feeders, artificial cover, and irrigation). Smaller home-range size and higher spatial overlap have previously been shown to be associated with high availability of anthropogenic food resources [65,68], and bobcats have larger home ranges when there are lower prey densities [64]. In our study, we observed bobcats capturing or carrying small mammals and birds that are common to urban areas, such as rats, squirrels, urban songbirds, and domestic ducks. Many parks and golf courses within our study site consisted of riparian areas that provided ideal cover for bobcat movement and hunting. More information on the diet of urban bobcats and prey availability would help elucidate whether prey abundance facilitates spatial overlap among bobcats.

Bobcats may also use different habitat types depending on their behavioral state, such as when they are foraging versus resting [69]. In such a mosaic of urban landscape cover, it would be informative to perform analyses, such as for RSF, based on active or inactive periods, and we suggest future habitat analyses consider bobcat activity patterns. We were unable to do that here because of the mismatch between the GPS-location schedule and their activity patterns. We had programmed the collars to collect more night locations because previous studies showed bobcats are more active overnight. In areas where mesopredators are released from human harvest pressure, they shift from nocturnal to diurnal activity patterns (e.g., [70]). However, bobcats remain nocturnal in other urban areas, likely to avoid other disturbance created by humans in urban environments [70,71]. Interestingly, bobcats in this system did not show differences in activity rates by time of day. It is unclear why this population differs from other urban areas, but it could be that bobcats are tracking activity of prey items or avoiding intraguild interactions. We detected free-roaming dogs (*Canis lupus familiaris*), coyotes, and raccoons (*Procyon lotor*) on camera traps within our study site [21]. Unique to this system relative to other urban studies is that we also detected feral pigs (*Sus scrofa*). Because it is evident that urban bobcats in DFW are active at all times of day and night, we suggest a different GPS-location program schedule for future studies and targeted collection information on the intraguild community.

Urbanization and loss of habitat is a leading threat to many large mammalian species, especially carnivores that often directly compete with humans for resources [72]. Understanding habitat use of urban bobcats will aid wildlife managers and urban decision-makers in continuing efforts to improve human–carnivore coexistence in urban areas. Findings from our study revealed bobcats in an urban landscape are selecting landscape features similar to bobcats in more natural areas. A population of bobcats in the heart of a dense major metropolitan area, such as DFW, provides optimistic possibilities for the potential of bobcats and other carnivores to thrive in an urban landscape with minimal conflict. Citizens recreating or living near areas associated with high probability of bobcat occurrence can be informed accordingly to enhance current preventative methods in minimizing perceived but often unsubstantiated fears about urban bobcats.

## Figures and Tables

**Table 1:** *P*-values from proximity analysis (Prox) in Program R at a range of distance thresholds for simultaneous GPS-collar locations of bobcats in the Dallas–Fort Worth metroplex, Texas, USA. There were no significant differences for age or sex of pairs nor were there differences in proximity relative to randomly generated paired locations, suggesting GPS-collared bobcats were not actively avoiding or attracted to one another.

Distance Threshold (m)	All Bobcats (*n* = 9)			Without Disperser (*n* = 8)		
	Age	Sex	Random	Age	Sex	Random
200	0.80	0.53	0.67	0.79	0.65	0.64
300	0.70	0.55	0.45	0.68	0.66	0.41
500	0.69	0.56	0.82	0.68	0.64	0.79
700	0.71	0.58	0.82	0.73	0.64	0.83
1000	0.85	0.36	0.83	0.86	0.45	0.82
1500	0.91	0.21	0.68	0.92	0.32	0.69
2000	0.73	0.14	0.74	0.73	0.28	0.26

## References

[1] Ripple W.J., Estes J.A., Beschta R.L., Wilmers C.C., Ritchie E.G., Hebblewhite M., Berger J., Elmhagen B., Letnic M., Nelson M.P. (2014). Status and ecological effects of the world’s largest carnivores. Science.

[2] Crooks K.R., Burdett C.L., Theobald D.M., Rondinini C., Boitani L. (2011). Global patterns of fragmentation and connectivity of mammalian carnivore habitat. Philos. Trans. R. Soc. Lond. B Biol. Sci..

[3] Di Minin E., Slotow R., Hunter L.T.B., Pouzols F.M., Toivonen T., Verburg P.H., Leader-Williams N., Petracca L., Moilanen A. (2016). Global priorities for national carnivore conservation under land use change. Sci. Rep..

[4] Loveridge A.J., Valeix M., Chapron G., Davidson Z., Mtare G., Macdonald D.W. (2016). Conservation of large predator populations: Demographic and spatial responses of African lions to the intensity of trophy hunting. Biol. Conserv..

[5] Ferreira A.S., Peres C.A., Bogoni J.A., Cassano C.R. (2018). Use of agroecosystem matrix habitats by mammalian carnivores (Carnivora): A global-scale analysis. Mamm. Rev..

[6] Athreya V., Odden M., Linnell J.D., Karanth K.U. (2011). Translocation as a tool for mitigating conflict with leopards in human-dominated landscapes of India. Conserv. Biol..

[7] Löe J., Röskaft E. (2004). Large carnivores and human safety: A review. AMBIO.

[8] Athreya V., Odden M., Linnell J.D., Krishnaswamy J., Karanth U. (2013). Big cats in our backyards: Persistence of large carnivores in a human dominated landscape in India. PLoS ONE.

[9] Bhatia S., Athreya V., Grenyer R., Macdonald D.W. (2013). Understanding the role of representations of human–leopard conflict in Mumbai through media-content analysis. Conserv. Biol..

[10] Singh P., Gopalaswamy A.M., Karanth K.U. (2010). Factors influencing densities of striped hyenas (*Hyaena hyaena*) in arid regions of India. J. Mammal..

[11] Abay G.Y., Bauer H., Gebrihiwot K., Deckers J. (2011). Peri-urban spotted hyena (*Crocuta crocuta*) in northern Ethiopia: Diet, economic impact, and abundance. Eur. J. Wildl. Res..

[12] Yirga G., Ersino W., De Iongh H.H., Leirs H., Gebrehiwot K., Deckers J., Bauer H. (2013). Spotted hyena (*Crocuta crocuta*) coexisting at high density with people in Wukro district, northern Ethiopia. Mammal. Biol..

[13] Boydston E.E., Kapheim K.M., Watts H.E., Szykman M., Holekamp K.E. (2003). Altered behaviour in spotted hyenas associated with increased human activity. Anim. Conserv..

[14] Benson J.F., Sikich J.A., Riley S.P. (2016). Individual and population level resource selection patterns of mountain lions preying on mule deer along an urban-wildland gradient. PLoS ONE.

[15] Gehrt S.D., Brown J.L., Anchor C. (2011). Is the urban coyote a misanthropic synanthrope? The case from Chicago. Cities Environ. CATE.

[16] Crooks K.R. (2002). Relative sensitivities of mammalian carnivores to habitat fragmentation. Conserv. Biol..

[17] Riley S.P., Sauvajot R.M., Fuller T.K., York E.C., Kamradt D.A., Bromley C., Wayne R.K. (2003). Effects of urbanization and habitat fragmentation on bobcats and coyotes in southern California. Conserv. Biol..

[18] Lombardi J.V., Comer C.E., Scognamillo D.G., Conway W.C. (2017). Coyote, fox, and bobcat response to anthropogenic and natural landscape features in a small urban area. Urban Ecosyst..

[19] Ruell E.W., Riley S.P., Douglas M.R., Pollinger J.P., Crooks K.R. (2009). Estimating bobcat population sizes and densities in a fragmented urban landscape using noninvasive capture–recapture sampling. J. Mammal..

[20] Tigas L.A., Van Vuren D.H., Sauvajot R.M. (2002). Behavioral responses of bobcats and coyotes to habitat fragmentation and corridors in an urban environment. Biol. Conserv..

[21] Young J.K., Golla J.M., Broman D., Blankenship T., Heilbrun R. (2019). Estimating density of an elusive carnivore in urban areas: Use of spatially explicit capture-recapture models for city-dwelling bobcats. Urban Ecosyst..

[22] Šálek M., Drahníková L., Tkadlec E. (2015). Changes in home range sizes and population densities of carnivore species along the natural to urban habitat gradient. Mammal. Rev..

[23] Burt W.H. (1943). Territoriality and home range concepts as applied to mammals. J. Mammal..

[24] Davison J., Huck M., Delahay R.J., Roper T.J. (2009). Restricted ranging behaviour in a high-density population of urban badgers. J. Zool..

[25] Harrison R.L. (1997). A comparison of gray fox ecology between residential and undeveloped rural landscapes. J. Wildl. Manag..

[26] Bateman P.W., Fleming P.A. (2013). Big city life: Carnivores in urban environments. J. Zool..

[27] Bailey T.N. (1974). Social organization in a bobcat population. J. Wildl. Manag..

[28] Larivière S., Walton L.R. (1997). Lynx rufus. Mammal. Spec..

[29] Anderson E.M. (1988). Effects of male removal on spatial distribution of bobcats. J. Mammal..

[30] Lovallo M.J., Anderson E.M. (1995). Range shift by a female bobcat (*Lynx rufus*) after removal of neighboring female. Am. Midl. Nat..

[31] Allen M.L., Wallace C.F., Wilmers C.C. (2015). Patterns in bobcat (*Lynx rufus*) scent marking and communication behaviors. J. Ethol..

[32] Benson J.F., Chamberlain M.J., Leopold B.D. (2004). Land tenure and occupation of vacant home ranges by bobcats (*Lynx rufus*). J. Mammal..

[33] Nielsen C.K., Woolf A. (2001). Spatial organization of bobcats (*Lynx rufus*) in southern Illinois. Am. Midl. Nat..

[34] López-Vidal J.C., Elizalde-Arellano C., Hernández L., Laundré J.W., González-Romero A., Cervantes F.A. (2014). Foraging of the bobcat (*Lynx rufus*) in the Chihuahuan Desert: Generalist or specialist?. Southwest. Nat..

[35] McCleery R.A. (2010). Urban mammals. Urban Ecosystem Ecology.

[36] Kertson B.N., Spencer R.D., Marzluff J.M., Hepinstall-Cymerman J., Grue C.E. (2011). Cougar space use and movements in the wildland–urban landscape of western Washington. Ecol. Appl..

[37] Gould F.W. (1975). The Grasses of Texas.

[38] Beltrán J.F., Tewes M.E. (1995). Immobilization of ocelots and bobcats with ketamine hydrochloride and xylazine hydrochloride. J. Wildl. Dis..

[39] Heilbrun R.D., Silvy N.J., Peterson M.J., Tewes M.E. (2006). Estimating bobcat abundance using automatically triggered cameras. J. Wildl. Manag..

[40] Elizalde-Arellano C., López-Vidal J.C., Hernández L., Laundré J.W., Cervantes F.A., Alonso-Spilsbury M. (2012). Home range size and activity patterns of bobcats (*Lynx rufus*) in the southern part of their range in the Chihuahuan Desert, Mexico. Am. Midl. Nat..

[41] D’Eon R.G., Delparte D. (2005). Effects of radio-collar position and orientation on GPS radio-collar performance, and the implications of PDOP in data screening. J. Appl. Ecol..

[42] ESRI (2013). ArcGIS Desktop: Release 10.1.

[43] Manly B.F.J., McDonald L.L., Thomas D.L., MacDonald T.L., Erickson W.P. (2002). Resource Selection by Animals. Statistical Design and Analysis for Field Studies.

[44] Benhamou S., Cornélis D. (2010). Incorporating movement behavior and barriers to improve kernel home range space use estimates. J. Wildl. Manag..

[45] Calenge C., Fortmann-Roe S. (2013). adehabitatHR: Home Range Estimation. R Package Version 0.4.7. http://cran.r-project.org/web/packages/adehabitatHR/.

[46] R Development Core Team (2013). R: A Language and Environment for Statistical Computing.

[47] Getz W.M., Wilmers C.C. (2004). A local nearest-neighbor convex-hull construction of home ranges and utilization distributions. Ecography.

[48] Lichti N.I., Swihart R.K. (2011). Estimating utilization distributions with kernel versus local convex hull methods. J. Wildl. Manag..

[49] Signer J., Balkenhol N. (2015). Reproducible home ranges (rhr): A new, user-friendly R package for analyses of wildlife telemetry data. Wildl. Soc. Bull..

[50] Homer C., Dewitz J., Yang L.M., Jin S., Danielson P., Xian G., Coulston J., Herold N., Wickham J., Megown K. (2015). Completion of the 2011 National Land Cover Database for the Conterminous United States—Representing a Decade of Land Cover Change Information. Photogramm. Eng. Remote Sens..

[51] Texas Water Development Board. https://www.twdb.texas.gov/mapping/gisdata.asp.

[52] Gillies C.S., Hebblewhite M., Nielsen S.E., Krawchuk M.A., Aldridge C.L., Frair J.L., Saher D.J., Stevens C.E., Jerde C.L. (2006). Application of random effects to the study of resource selection by animals. J. Anim. Ecol..

[53] Burnham K.P., Anderson D.R. (2002). Model Selection and Multimodel Inference: A Practical Information-Theoretic Approach.

[54] Avgar T., Lele S.R., Keim J.L., Boyce M.S. (2017). Relative selection strength: Quantifying effect size in selection inference. Ecol. Evol..

[55] Bertrand M.R., DeNicola A.J., Beissinger S.R., Swihart R.K. (1996). Effects of parturition on home ranges and social affiliations of female white-tailed deer. J. Wildl. Manag..

[56] Long J.A. wildlifeDI—A Suite of R Tools for Exploring Dynamic Interaction Patterns in Wildlife Telemetry Data Version 0.1 (2014). http://citeseerx.ist.psu.edu/viewdoc/summary?doi=10.1.1.565.460.

[57] Riley S.P. (2006). Spatial ecology of bobcats and gray foxes in urban and rural zones of a national park. J. Wildl. Manag..

[58] Ordeñana M.A., Crooks K.R., Boydston E.E., Fisher R.N., Lyren L.M., Siudyla S., Haas C.D., Harris S., Hathaway S.A., Turschak G.M. (2010). Effects of urbanization on carnivore species distribution and richness. J. Mammal..

[59] Lawhead D.N. (1984). Bobcat *Lynx rufus* home range, density and habitat preference in south-central Arizona. Southwest. Nat..

[60] Hamilton D.A. (1982). Ecology of the Bobcat in Missouri. Master’s Thesis.

[61] Zezulak D.S., Schwab R.G. (1979). A comparison of density, home range, and habitat utilization of bobcat populations at Lava Beds and Joshua Tree National Monuments, California. Proc. 1979 Bobcat Res. Conf. National Wildl. Fed. Sci. Tech. Series.

[62] Conner L.M., Chamberlain M.J., Leopold B.D. (2001). Bobcat home range size relative to habitat quality. Proc. Annu. Conf. SEAFWA.

[63] Ruell E.W., Riley S.P.D., Douglas M.R., Antolin M.F., Pollinger J.R., Tracey J.A., Lyren L.M., Boydston E.E., Fisher R.N., Crooks K.R. (2012). Urban habitat fragmentation and genetic population structure of bobcats in coastal southern California. Am. Midl. Nat..

[64] Blankenship T.L. (2000). Ecological Response of Bobcats to Fluctuating Prey Populations on the Welder Wildlife Foundation Refuge. Ph.D. Dissertation.

[65] Fedriani J.M., Fuller T.K., Sauvajot R. (2001). Does availability of anthropogenic food enhance densities of omnivorous mammals? An example with coyotes in southern California. Ecography.

[66] Poessel S.A., Burdett C.L., Boydston E.E., Lyren L.M., Alonso R.S., Fisher R.N., Crooks K.R. (2014). Roads influence movement and home ranges of a fragmentation-sensitive carnivore, the bobcat, in an urban landscape. Biol. Conserv..

[67] Cuo L., Lettenmaier D.P., Alberti M., Richey J.E. (2009). Effects of a century of land cover and climate change on the hydrology of the Puget Sound basin. Hydrol. Process..

[68] Cresswell W.J., Harris S. (1988). Foraging behaviour and home-range utilization in a suburban badger (*Meles meles*) population. Mammal. Rev..

[69] Abrahms B., Jordan N.R., Golabek K.A., McNutt J.W., Wilson A.M., Brashares J.S. (2016). Lessons from integrating behaviour and resource selection: Activity-specific responses of African wild dogs to roads. Anim. Conserv..

[70] Kitchen A.M., Gese E.M., Schauster E.R. (2000). Changes in coyote activity patterns due to reduced exposure to human persecution. Can. J. Zool..

[71] Smith J.A., Suraci J.P., Clinchy M., Crawford A., Roberts D., Zanette L.Y., Wilmers C.C. (2017). Fear of the human ‘super predator’ reduces feeding time in large carnivores. Proc. R. Soc. B Biol. Sci..

[72] Breck S.W., Poessel S.A., Mahoney P., Young J.K. (2019). The intrepid urban coyote: A comparison of bold and exploratory behavior in coyotes from urban and rural environments. Sci. Rep..

